# Abnormal skin in toe webs is a marker for abnormal glucose metabolism. A cross-sectional survey among 1,849 adults in Finland

**DOI:** 10.1038/s41598-017-09354-3

**Published:** 2017-08-22

**Authors:** Suvi-Päivikki Sinikumpu, Juha Auvinen, Jari Jokelainen, Laura Huilaja, Katri Puukka, Aimo Ruokonen, Sirkka Keinänen-Kiukaanniemi, Kaisa Tasanen, Markku Timonen

**Affiliations:** 1 PEDEGO Research Unit, University of Oulu, Department of Dermatology and Medical Research Center Oulu, Oulu University Hospital, P.O. Box 20, Oulu, 90029 Finland; 2 Unit of General Practice, Oulu University Hospital, P.O. Box 10, Oulu, 90029 Finland; 3 Centre for Life Course Health Research, University of Oulu, P.O. Box 5000, Oulu, 90014 Finland; 4 NordLab Oulu, Department of Clinical Chemistry and Medical Research Center Oulu, University Hospital of Oulu, P.O. Box 500, Oulu, 90029 Finland

## Abstract

Diabetes is undiagnosed disease and easy screening tools for it are warranted. Because foot complications are usual in diabetes, we aimed to test hypothesis that skin abnormalities are found already from patients who are not aware of having diabetes, by studying the possible association between unhealthy toe web skin and abnormal glucose metabolism. 1,849 cases without previously diagnosed diabetes participated to the 46-year follow-up study of the Northern Finland Birth Cohort. A skin investigation was performed for all, and abnormal skin findings in toe web spaces were taken as explanatory variables. Abnormal glucose tolerance was the main outcome and it was tested with an oral glucose tolerance test (OGTT), glycosylated haemoglobin fraction (HbA_1c_) Values are numbers (percentages) of sub and fasting blood glucose. The participants who had any abnormal skin findings in toe webs were associated with 2.5-fold (OR 2.5, 95% CI 1.3–4.9) and 6-fold (OR 6.2, 1.4–27.6) increased risk of having previously undiagnosed diabetes detected by a 2-hour OGTT and HbA_1c_, respectively. The predictive power of toe web findings was comparable with FINDRISC score. Abnormal skin findings in the toe webs show increased risk of occult diabetes, and may, thus serve as an additional sign of undiagnosed diabetes.

## Introduction

Disrupted micro- and macrovascular architecture caused by abnormal glucose metabolism may result in increased complications in the feet of diabetes patients^1^. Foot complications are one of the most common reasons for hospitalization of patients with diabetes^2, 3^. Among these complications, diabetic ulcers are the most common^4^, presenting in 2–5% of patients every year and this complication is seen in 15–25% of patients with diabetes at some point during their disease duration^5–7^. Diabetic ulcer precedes lower limb amputation in 80% of cases^6^ and gangrene, cellulitis and other skin infections can be life threatening^6, 8, 9^.

A lot of work has been done to prevent diabetic foot complications, e.g. by means of the National Institute for Health and Clinical Excellence (NICE) guidelines for diabetic foot problems. However, given that half of all adult patients with diabetes are still not diagnosed^1^, there is an unmet need not only for secondary prevention of complications but also for early detection and primary prevention of diabetes itself. Diabetes Risk Scores such as FINDRISC questionnaire is one of those tools that has been utilized to identify individuals at high risk of diabetes^10^. Based on the high incidence of foot problems in diabetes patients per se it is reasonable to hypothesize that abnormalities of the foot skin might also be found in individuals who are not yet aware of having diabetes or prediabetes stage. We tested this hypothesis – by first performing comprehensive clinical investigations of foot skin and oral glucose tolerance tests (OGTT), and recording fasting plasma glucose (FPG) and glycosylated haemoglobin fraction (HbA_1c_) in birth cohort participants - to determine any association between skin findings in the toe web spaces and previously undiagnosed diabetes or prediabetes.

## Results

### Characteristics of study population and toe web findings

A total of 3,181 cohort members were invited to participate in the study; of these 1,930 (60.7%) agreed and were examined by the dermatologists. All study participants were 45–46 years old at the time of data collection; 53.6% were females. A total of 81 participants (4.2%) had previously diagnosed diabetes (prDM) and they were excluded from the present study, and, consequently the rest study cases formed our final study population (N = 1,849). FPG was recorded in 1,804 (97.6%) of participants (data missing from 45 [2.4%]) and HbA_1c_ data were available from 1,838 (99.4%) (data missing from 11 [0.6%]). Altogether, 1,571 (85.0%) attended OGTT, and 1,554 (84.0%) completed the test. (S1, Table 1). The reasons for missing laboratory data were that all study cases did not attend the OGTT or that some of blood samples or their analysis were not eligible.

**Table 1:** The characteristics of the cohort study population concerning skin findings in toe web spaces, glucose metabolism and confounding factors according to sex. Values are numbers (percentages) of subjects unless stated otherwise.

	Male	Female	P value*
Variables			
Skin changes in toe web spaces:			<0.001
No	545 (63.3)	812 (82.2)	
Yes	316 (36.7)	176 (17.8)	
OGTT status:			<0.001
Normal glucose tolerance	542 (74.3)	714 (86.5)	
Impaired fasting glycemia	92 (12.6)	29 (3.5)	
Impaired glucose tolerance	73 (10.0)	61 (7.4)	
Screen-detected diabetes	22 (3.0)	21 (2.5)	
OGTT 0-h glucose (mmol/L)^†^:	5.7 (0.6)	5.3 (0.6)	<0.001
OGTT 0-h glucose^‡^:			<0.001
Normal	593 (80.5)	773 (92.7)	
Elevated	127 (17.2)	50 (6.0)	
Diabetic	17 (2.3)	11 (1.3)	
OGTT 2-h glucose (mmol/L)^†^:	6.0 (1.6)	5.9 (1.6)	0.676
OGTT 2-h glucose (mmol/L)^‡^:			0.083
Normal	644 (88.3)	750 (90.9)	
Elevated	77 (10.6)	62 (7.5)	
Diabetic	8 (1.1)	13 (1.6)	
Fasting plasma glucose (mmol/L)^†^:	5.6 (0.7)	5.2 (0.5)	<0.001
Fasting plasma glucose (mmol/L)^§^:			<0.001
Normal	698 (83.4)	909 (94.0)	
Elevated	121 (14.5)	51 (5.3)	
Diabetic	18 (2.2)	7 (0.7)	
Fasting HbA_1C_ (mmol/mol)^†^:	36.1 (5.2)	35.0 (3.8)	<0.001
Fasting HbA_1C_ (%)^†^:	5.5 (0.5)	5.4 (0.4)	<0.001
Fasting HbA_1C_ (%)^‖^:			<0.001
Normal	632 (73.7)	794 (80.9)	
Elevated	214 (25.0)	183 (18.7)	
Diabetic	11 (1.3)	4 (0.4)	
Education:			0.276
Basic/Secondary	539 (62.6)	593 (60.0)	
Tertiary	322 (37.4)	395 (40.0)	
Smoking status^#^:			<0.001
Never smoked	392 (47.4)	553 (58.0)	
Former smokers (>6kk)	232 (28.1)	217 (22.8)	
Former smokers (<6kk)	18 (2.2)	12 (1.3)	
Current smokers	185 (22.4)	171 (17.9)	
Physical activity^#^:			<0.001
Inactive	157 (19.0)	209 (21.9)	
Lightly active	324 (39.3)	364 (38.1)	
Active	301 (36.5)	365 (38.2)	
Very active	43 (5.2)	17 (1.8)	
Body mass index (kg/m^2^)^†^:	27.1 (4.0)	26.4 (5.2)	<0.001
Body mass index (kg/m^2^):^”^			<0.001
Underweight	2 (0.2)	6 (0.6)	
Normal	266 (30.9)	468 (47.5)	
Overweight	431 (50.1)	303 (30.7)	
Obese	134 (15.6)	142 (14.4)	
Severely Obese	28 (3.3)	67 (6.8)	

*The Mann-Whitney U-test was used when testing continuous variables and Chi-Square test or Fisher exact test for categorical variables. ^†^Values are mean (standard deviation, SD). ^‡^OGTT: 1,571 study cases first attended and 1,554 of them completed OGTT. OGTT was not conducted if FPG was <8.0 mmol/l just before the test (n = 11). OGTT 0-h or OGTT 2-h was not eligible in six (n = 6) study cases. N = 278 did not attend the OGTT. ^§^Data of FPG was missing from n = 45. ^§^Data of HbA1c was missing from n = 11. ^#^Data of smoking status and physical activity was missing from n = 69 (The reason was missing data was that all study cases did not fill health questionnaires). ^”^Data of body mass index was missing from n = 2 (The reason for missing data was that all study cases did not come to the weighing).

Abnormal skin features were found in the toe web spaces of 26.6% (N = 492/1,849) of the participants. They were found significantly more often in males (36.7%, N = 316) compared to females (17.8%, N = 176) (P < 0.001). (Tables 1 and 2).

**Table 2:** Skin findings in toe web spaces and its associative factors.

	Skin findings in toe web spaces		P value*
	No	Yes	
Variables			
Sex:			<0.001
Male	545 (40.2)	316 (4.2)	
Female	812 (59.8)	176 (35.8)	
OGTT status:			<0.001
Normal glucose tolerance	954 (83.1)	302 (74.4)	
Impaired fasting glycemia	79 (6.9)	42 (10.3)	
Impaired glucose tolerance	96 (8.4)	38 (9.4)	
Screen detected diabetes	19 (1.7)	24 (5.9)	
OGTT 0-h glucose(mmol/L)^†^:	5.5 (0.5)	5.7 (0.8)	<0.001
OGTT 0-h glucose^‡^:			<0.001
Normal	1033 (89.1)	333 (81.0)	
Elevated	114 (9.8)	63 (15.3)	
Diabetic	13 (1.1)	15 (3.6)	
OGTT 2-h glucose (mmol/L)^†^:	5.9 (1.5)	6.0 (1.8)	0.978
OGTT 2-h glucose^‡^:			0.003
Normal	1043 (90.6)	351 (87.1)	
Elevated	99 (8.6)	40 (9.9)	
Diabetic	9 (0.8)	12 (3.0)	
Fasting plasma glucose (mmol/L)^†^:	5.4 (0.6)	5.6 (0.8)	<0.001
Fasting plasma glucose (mmol/L)^§^:			0.001
Normal	1196 (90.5)	411 (85.3)	
Elevated	114 (8.6)	58 (12.0)	
Diabetic	12 (0.9)	13 (2.7)	
Fasting HbA_1C_ (mmol/mol)^†^:	35.2 (4.3)	36.4 (5.1)	<0.001
Fasting HbA_1C_ (%)^†^:	5.4 (0.4)	5.5 (0.5)	<0.001
Fasting HbA_1C_ (%)^‖^:			<0.001
Normal	1077 (79.8)	349 (71.5)	
Elevated	267 (19.8)	130 (26.6)	
Diabetic	6 (0.4)	9 (1.8)	
Education:			0.005
Basic/Secondary	804 (59.2)	328 (66.7)	
Tertiary	553 (40.8)	164 (33.3)	
Smoking status^#^			0.002
Never smoked	713 (54.3)	232 (49.6)	
Former smokers (>6kk)	339 (25.8)	110 (23.5)	
Former smokers (<6kk)	25 (1.9)	5 (1.1)	
Current smokers	235 (17.9)	121 (25.9)	
Physical activity^#^			0.029
Inactive	258 (19.7)	108 (22.8)	
Lightly active	509 (38.9)	179 (37.8)	
Active	504 (38.6)	162 (34.2)	
Very active	36 (2.8)	24 (5.1)	
Body mass index (kg/m^2^)^†^:	26.3 (4.5)	27.9 (5.1)	<0.001
Body mass index (kg/m^2^):^”^			<0.001
Underweight	6 (0.4)	2 (0.4)	
Normal	590 (43.5)	144 (29.3)	
Overweight	521 (38.5)	213 (43.3)	
Obese	181 (13.4)	95 (19.3)	
Severely Obese	57 (4.2)	38 (7.7)	

Values are numbers (percentages) of subjects unless stated otherwise. *The Mann-Whitney U-test for used when testing continuous variables and Chi-Square test or Fisher exact test for categorical variables. ^†^Values are mean (standard deviation, SD). ^‡^OGTT: 1,571 study cases first attended and 1,554 of them completed OGTT. OGTT was not conducted if FPG was <8.0 mmol/l just before the test (n = 11). OGTT 0-h or OGTT 2-h was not eligible in six (n = 6) study cases. N = 278 did not attend the OGTT. ^§^Data of FPG was missing from n = 45. ^**ǁ**^Data of HbA1c was missing from n = 11. ^#^Data of smoking status and physical activity was missing from n = 69 (The reason was missing data was that all study cases did not fill health questionnaires). ^”^Data of body mass index was missing from n = 2 (The reason for missing data was that all study cases did not come to the weighing).

### Glucose metabolism

Among study participants FPG was elevated (6.1–6.9 mmol/L) in 9.5% (N = 172/1,804) and 1.2% (N = 21/1,804) presented diabetic value of ≥7.0 mmol/L. HbA_1c_ was elevated (5.7–6.4%) in 21.6% of the study cases (N = 397/1,838) and it was at diabetic level (≥6.5%) in 0.8% (N = 15/1,838). According to OGTT test, 2.8% (N = 43/1,554) had screen-detected diabetes (SDM), impaired fasting glycaemia (IFG) was found in 7.9% (N = 121/1,554) of the study participants and 8.6% (N = 134/1,554) had impaired glucose tolerance (IGT).

### Association of skin findings in toe web spaces and glucose metabolism

**Figure 1:**
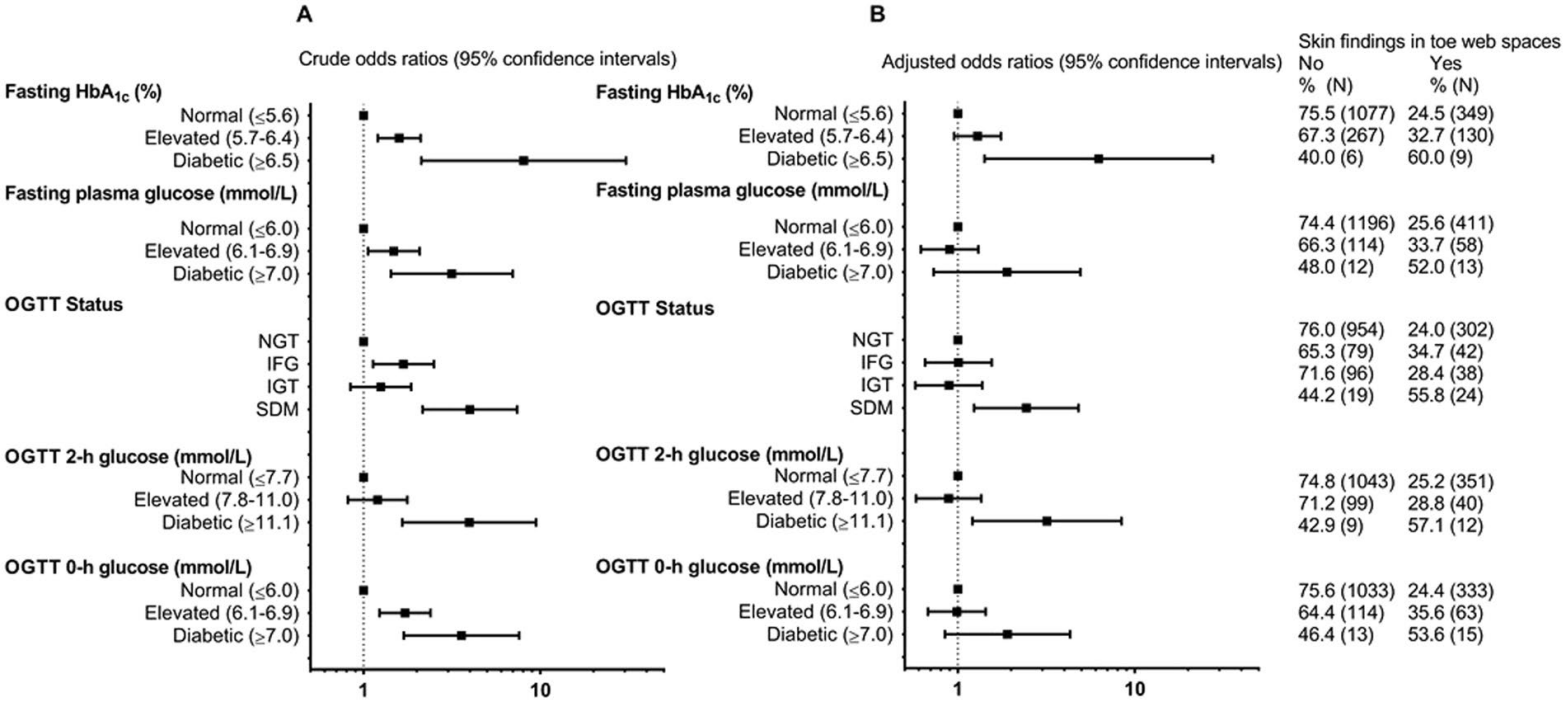
Forest plot of risk for abnormal glucose metabolism in subjects with skin changes in toe web spaces. Multinomial logistic regression analysis, crude (**A**) and adjusted (**B**) odds ratios and their 95% confidence intervals are presented. Adjustments are made for body mass index, smoking, physical activity, education and sex. OGTT = oral glucose tolerance test, NGT = normal glucose tolerance, IFG = impaired fasting glycaemia, IGT = impaired glucose tolerance, SDM = screen-detected diabetes. Skin changes in toe web spaces = maceration, scales, vesicles or localized erythema were systemically recorded as abnormal cutaneous findings of the toe web spaces.

Subjects with abnormal skin findings in the toe webs had a 2.5-fold greater risk of having SDM by the OGTT than those without abnormal findings (adjusted odds ratio (OR) 2.48, 95% confidence interval (CI) 1.3–4.91; P = 0.01). The corresponding separate analyses with regard to 0-h (fasting) and 2-h values in 2-h OGTT are presented in Fig. 1. Diabetic HbA_1c_ levels were also more common in participants with abnormal toe web findings (1.8%, N = 9/492), compared to those with healthy skin (0.4%, N = 6/1,357) (adjusted OR 6.2, 95% CI 1.4–27.6; P < 0.001). The association of skin changes in toe web spaces between IFG and IGT did not reach the level of statistical significance after the adjusting for confounding variables (adjusted OR 1.01, 95% CI 0.65–1.55; P = 0.98 and adjusted OR 0.89, 95% CI 0.58–1.37; P = 0.60 for IFG and IGT, respectively) (Table 2) (Fig. 1).

### The predictive power of toe web space finding

Multivariate logistic regression analyses demonstrated that both FINDRISC score and toe webs finding were independent risk factors to predict diabetes risk (i.e. SDM) in our study population (adjusted OR for FINDRISC was 1.5, 95% CI 1.3–1.6 and for toe web finding 2.3, 95% CI 1.2–4.2; P < 0.01 for both). When the toe web finding was added to the original FINDRISC model^10^, the β-coefficient for toe web finding was 0.82, which corresponds to a score of 3 in the FINDRISC test^10^. The AUC, the area under the receiver operating characteristic (ROC) curve, was 0.834 for the original FINDRISC score and 0.839 for the risk score modified with toe web finding.

## Discussion

The central finding of our general population-based study was that, after adjusting for confounding variables, abnormal skin findings in the toe web spaces were associated with an elevated risk of having SDM. The corresponding risk was 2.5-fold, and even over 6-fold higher when elevated 2-h glucose, and HbA_1c_, respectively, were used as a definition of SDM.

There are several clinical findings, such as high blood pressure and obesity, that are associated with diabetes mellitus and their appearance may, therefore, result in suspicion of the diabetes^11, 12^. Further, impaired reflexes^13^, vision^14^, increased urinating^15^ and cardiac ischemia^16^ are other common symptoms that can precede a diagnosis of diabetes. To best of our knowledge, this is the first study to investigate the association between skin changes in the toe web spaces and prediabetes or undiagnosed diabetes using clinical skin examinations and an OGTT as well as blood tests.

Significant effort has been made to develop practical, easy, fast and non-invasive screening tools for to identify persons who are unknowingly at high risk of type 2 diabetes^17^. Of those, one of the most widely used screening tools among Caucasians is FINDRISC questionnaire which has been described to identify up to 66% of males and 70% of females of previously undiagnosed patients with type 2 diabetes^10^. In order to test the predictive power of abnormal toe webs finding we compared it to FINDRISC score. These analyses revealed that toe webs finding is an independent risk factor to predict diabetes risk: the β-coefficient for the toe web finding was corresponding to a score of 3 in the FINDRISC test and is therefore comparable to body mass index or waist circumference as a risk factor. However, our variable did not add much to the predictive power of the statistical model, since the AUC for the FINDRISC modified with the toe web finding was only slightly higher than that of the original FINDRISC score. Albeit toe web finding is an independent risk factor in risk score, its clinical importance at population level requires future studies.

There are some theoretical explanations for the overrepresentation of skin changes in abnormal glucose metabolism; both hyperglycaemia and decreased insulin signal affect skin function^18^. Insulin is an important growth factor of keratinocytes and is involved in their proliferation, migration and differentiation^19^. When the insulin balance is disrupted, the functions of keratinocytes are impaired, the epidermal barrier is disrupted and wound healing worsens^20^. In hyperglycaemia the concentration of cutaneous glucose increases and the pH of intertriginous regions of the skin surface rises, favouring colonization of microbes. Furthermore, diabetes causes decreases in sebaceous gland activity and skin hydration resulting in dry skin that is still more vulnerable to infections^21^. Finally, other complications associated with diabetes, such as vascular sclerosis and neuropathy also increase the risk of skin complications^20^. Fungal skin infections have been reported to be present up to 30–80% of people with diabetes, and might be due to these earlier mentioned theoretical explanations^22–27^. Tinea pedis, which is the most common fungal skin infection in general and in patients with diabetes, is typically found in the toe web spaces between the fourth and fifth toes and is characterized by maceration, peeling or erythema^28^. Consequently, our findings in toe web spaces of subjects who did not know as having diabetes might be explained mainly because of tinea pedis. The early detection of diabetes and subsequent foot care is important in order to prevent complications of diabetes. These complications may initially manifest as interdigital toe web maceration that can progress towards fulminant infections like erysipelas, cellulitis, sepsis and osteomyelitis^29–32^ and even to diabetic ulcer^6^.

Toe web skin and glucose metabolism were investigated in a large general population. Skin examination was performed for nearly 2,000 study participants by a specialist in dermatology or by an experienced resident instead of self-reporting. Maceration, vesicles or localized erythema in the toe webs were recognised as abnormalities considering that they are findings that can easily be found in the adult population by subjects themselves or by an expert other than a dermatologist. No specific instruments or laboratory tests such as fungal culture were needed: a visual investigation was sufficient to determine unhealthy toe webs. This emphasises the importance of our primary finding, because it means that self-determined problems in the toe web spaces may function as the first signal of undiagnosed diabetes thus enhancing the early detection of diabetes. At 60.7%, the participation rate was satisfactorily high. Finally, glucose metabolism was tested with HbA_1c_ and fasting glucose for nearly 2,000 and with OGTT for nearly 1,600 study cases.

One weakness of our study was that we did not take any culture samples of toe web spaces and it was impossible to determine whether such samples would have revealed fungal, yeast or bacterial infection in the skin. Nevertheless, visible risk factors (toe web space abnormalities) are more important from the prevention point of view and therefore, when designing our study, we gave preference to clinical status over culture as our candidate sign of prediabetes or diabetes. Abnormalities in toe web spaces are usually easy to notice. However, the signs can be so slight that they are easily ignored by individuals themselves or even by their doctors, and their clinical importance can be overlooked.

According to the findings of our study, unhealthy toe web spaces could also be taken into account to enhance the detection of diabetes; maceration, scales, vesicles or localized erythema in the toe web spaces may be one sign of abnormal glucose metabolism in middle-aged people who are not aware that they have diabetes. Our population was predominantly made up of subjects in early middle-aged, and since, the onset of diabetes usually occurs later in adulthood, the association between abnormal toe web space findings and decreased glucose tolerance should be evaluated as a future research in older people.

## Research Design and Methods

### Study design and population

This is a cross-sectional general population study that belongs to the Northern Finland Birth Cohort 1966 (NFBC1966), which is a longitudinal research program. NFBC1966 initially included all 12,058 children in the two northernmost provinces in Finland whose expected days of birth were between 1^st^ of January and 31^st^ of December 1966. The whole cohort has been evaluated regularly since birth by means of health questionnaires and clinical examinations.

When the NFBC1966 study participants reached the age of 46 years, all those living within 100 km of the centre of the city of Oulu (N = 3,181) were invited to participate in a multidisciplinary health study, which included comprehensive clinical health examination and questionnaires^33^. This study included a clinical skin examination and detailed inspection of feet, extraction of fasting blood samples and, on a separate day, a 2-hour OGTT. Data were collected between April 2012 and May 2013.

### Clinical skin examination

**Figure 2:**
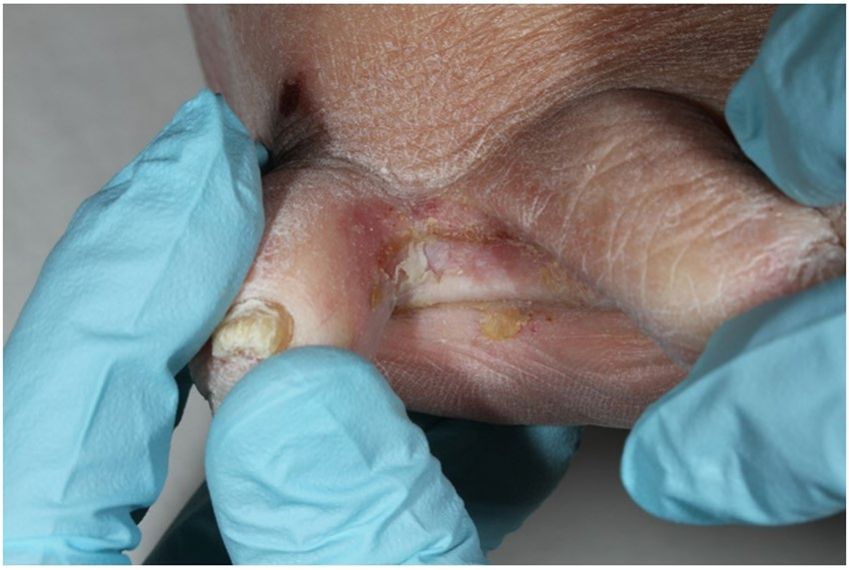
Abnormal skin findings in toe web space; maceration, scales and erythema.

A comprehensive dermatological examination was performed for 1,930 participants by a dermatologist or an experienced resident^33^. This full skin examination also included a detailed inspection of the toe web spaces and the skin surrounding them to detect any sign of abnormal findings. In particular maceration, scales, vesicles or localized erythema were systemically recorded as abnormal cutaneous findings of the toe web spaces. (Fig. 2).

### Glucose metabolism

In connection with clinical health examination, venous blood samples were used to determine fasting plasma glucose and HbA_1c_ –values of every eligible participant. Blood samples were taken at the cohort laboratory between 7:00 and 11:00 a.m. after an over-night fast. Patients whose fasting plasma glucose was <8.0 mmol/L and had no previous diagnosis of diabetes underwent an OGTT with a 75 g glucose load, after which a 2-hour plasma sample was collected. Previously diagnosed diabetes (PrDM) was defined according to self-reported diagnoses and medications, hospital outpatient and inpatient registers and medication registers from the Social Insurance Institution of Finland. A total of 81 participants had prDM and they were excluded from the study. The concentration of HbA_1c_ and the concentration of total haemoglobin were measured by immunochemical assay method. Glucose were analysed using an enzymatic hexokinase/glucose-6-phosphate dehydrogenase method. (both method: Advia 1800; Siemens Healthcare Diagnostics Inc., Tarrytown, Ny, USA). The samples were analysed in NordLab Oulu, a testing laboratory (T113) accredited by Finnish Accreditation Service (FINAS) (EN ISO 15189).

Glucose tolerance status was classified according to World Health Organization (WHO) criteria as follows:

- normal glucose tolerance (NGT) was defined as having a FPG level <6.1 mmol/L and a 2-hour post-load glucose level <7.8 mmol/L
- IGT was defined by a FPG level <7.0 mmol/L and a 2-hour glucose level of 7.8–11.1 mmol/L
- IFG was defined by a FPG level between 6.1- and 6.9 mmol/L and a 2-hour glucose level <7.8 mmol/L
- SDM was defined by either a FPG level ≥7.0 mmol/L or a 2-hour glucose level ≥1.1 mmol/L.

HbA_1C_ was defined as normal when <5.7%, elevated when 5.7–6.4% and diabetic when ≥6.5% according to American Diabetes Association (ADA).

### Confounding factors

Body mass index, smoking, leisure time physical activity and socioeconomic status were considered as potentially confounding factors in the analyses because they have all been associated with skin changes and abnormal glucose metabolism^33–40^.

Body mass index was determined according to the measured weight (kg) and height. Study participants were classified into five groups according to body mass index: Underweight <18.5; normal 18.5–25; overweight 25–30; obese 30–35 and severely obese >35.

Smoking status was reported in postal questionnaires at the age of 46 years using the following questions: 1) Have you ever smoked? 2) Have you ever smoked regularly, almost daily for at least a year? 3) Do you currently smoke? 4) When was the last time you smoked? According to the answers the respondents were divided into four groups: current smokers (those who smoked regularly and who had smoked in the last month); former smokers <6 months (those who had smoked regularly but had quit smoking less than six months ago), former smokers >6 months (those who had smoked but had quit more than six months ago), and 4) never smoked (those who had never smoked regularly for at least a year).

Leisure time physical activity was self-reported. Reporting concerned the frequency of participation to physical and recreational activities in leisure time and the study cases were classified into four groups: Inactive (those who preferred to stay indoors reading or watching television and did not like sports much); Lightly active (those who exercised at least four hours per week e.g. by walking, cycling, fishing); Active (those who liked fitness training and had e.g. running, swimming or skiing as a regular sport activity for at least two hours per week); Very active (those who exercised several hours per week by running, practicing orienteering or playing ball games.

Socioeconomic status was categorised on education level, because that has been defined as the most specific indicator of socioeconomic status^41^. Data concerning education were obtained from the National Education Register and were supplemented by self-reported questionnaires regarding personal educational history^42^.

### Statistical analyses

The main outcome measures were OGTT status (NGT, IFG, IGT, SDM or prDM), FPG and HbA_1c_ while skin findings in toe web space (normal vs. abnormal) was an explanatory variable. The skin was defined as abnormal if at least one of the following was found: maceration, scales, vesicles or localized erythema. Distributions of continuous variables were expressed as mean and standard deviation (SD), and categorical variables as proportions. Continuous variables were tested using the Mann-Whitney U-test. The Chi-Square test or Fisher’s exact test were used for categorical variables, as appropriate. The risk factors associated with abnormal glucose metabolism in participants (examined by OGTT, FPG and HbA_1c_) were analysed using a multivariate multinomial logistic regression technique. Skin findings in toe web spaces were analysed for correlation with abnormal glucose metabolism. Adjustments were made for smoking, physical activity, education, sex and body mass index. An established clinical FINDRISC score for type 2 diabetes was chosen as a reference tool against findings in toe web spaces. The logistic regression analysis was used to evaluate the risk for diabetes (i.e. SDM) both with FINDRISC score and with toe web finding. The FINDRISC risk score was modified by adding toe web finding as an additional variable into the FINDRISC model and logistic regression analyses was performed according to the methodology in the original publication by Lindström and Tuomilehto^10^ in order to test if toe web finding gives predictive power to this establish clinical risk score. The area under the receiver operating characteristic (ROC) curve, known as the AUC, was defined for FINDRISC and for risk score modified with toe web finding. ORs (crude and adjusted) with related 95% CI were reported as measures of association. All analyses were performed with the statistical package SAS v.9.4 (SAS Institute, Cary, Northern Canada, USA) and two-tailed p-values < 0.05 were considered to be statistically significant.

### Ethical aspects

The Ethical Committee of the Northern Ostrobothnia Hospital District approved the study (§94/2011), which was performed according to the principles of the Helsinki Declaration of 1983.

The participants took part on a voluntary basis and signed their informed consent. The data were handled on a group level only, personal information being replaced by identification codes resulting in complete anonymity.

### Data availability

All data generated or analysed during this study are included in this published article (and its Supplementary Information files).

## Supplementary information

Supplementary Information
