# Supplementary material for: Abnormal skin in toe webs is a marker for abnormal glucose metabolism. A cross-sectional survey among 1,849 adults in Finland
Source: Sci Rep. 2017 Aug 22;7:9125. doi: 10.1038/s41598-017-09354-3 (PMC5567349; doi:10.1038/s41598-017-09354-3)

**Abnormal skin in toe webs is a marker for abnormal glucose metabolism. A cross-sectional survey among 1,849 adults in Finland**

Suvi-Päivikki Sinikumpu^1^, Juha Auvinen^2^, Jari Jokelainen^2^, Laura Huilaja^1^, Katri Puukka^3^., Aimo Ruokonen^3^, Sirkka Keinänen-Kiukaanniemi^2^, Kaisa Tasanen^1^, Markku Timonen^2^

^1^PEDEGO Research Unit, University of Oulu; Department of Dermatology and Medical Research Center Oulu, Oulu University Hospital P.O. Box 20, 90029 Oulu University Hospital, Oulu, Finland

^2^Center for Life Course Health Research, Faculty of medicine University of Oulu and Oulu University Hospital, Oulu, Finland

^3^NordLab Oulu, Medical Research Center Oulu, Oulu University Hospital and Department of Clinical Chemistry, University of Oulu, Finland

*Corresponding author*: Suvi-Päivikki Sinikumpu, [suvi-paivikki.sinikumpu@oulu.fi](mailto:suvi-paivikki.sinikumpu@oulu.fi)

Department of Dermatology, Oulu University Hospital, Oulu, Finland

P.B.Box 20, FIN-90029 OYS, FINLAND;

tel.+35883152011, fax +35883153135


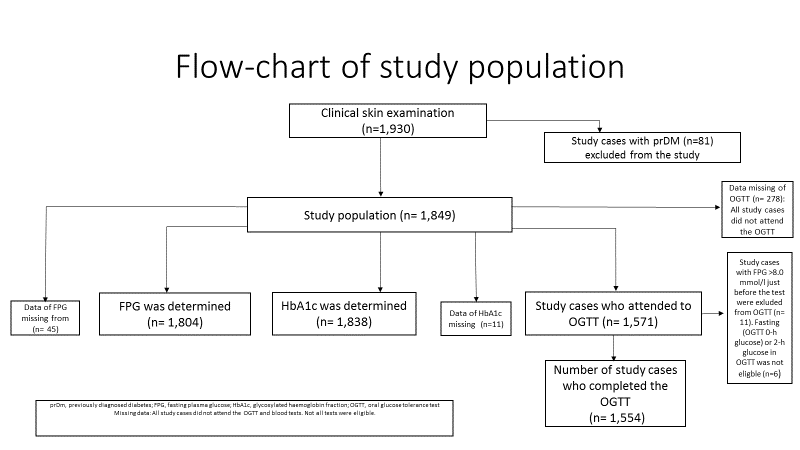

Supplement: Supplementary file 1 — Supplementary Information [file 41598_2017_9354_MOESM1_ESM.doc]
